# Bridging Hepatitis C Care Gaps: A Modeling Approach for Achieving the WHO’s Targets in Ontario, Canada

**DOI:** 10.3390/v16081224

**Published:** 2024-07-31

**Authors:** Yeva Sahakyan, Aysegul Erman, William W. L. Wong, Christina Greenaway, Naveed Janjua, Jeffrey C. Kwong, Beate Sander

**Affiliations:** 1 Toronto Health Economics and Technology Assessment Collaborative (THETA), University Health Network, Toronto, ON M5G 2C4, Canada; yeva.sahakyan@uhn.ca (Y.S.); aysegul.erman@mail.utoronto.ca (A.E.); 2 ICES, Toronto, ON M4N 3M5, Canada; 3 School of Pharmacy, University of Waterloo, Kitchener, ON N2G 1C5, Canada; william.wl.wong@uwaterloo.ca; 4 Division of Infectious Diseases, Jewish General Hospital, McGill University, Montreal, QC H3A 0G4, Canada; ca.greenaway@mcgill.ca; 5 British Columbia Centre for Disease Control (BCDC), Vancouver, BC V5Z 4R4, Canada; naveed@uab.edu; 6 Department of Family and Community Medicine, Temerty Faculty of Medicine, University of Toronto, Toronto, ON M5S 1A8, Canada; jeff.kwong@utoronto.ca; 7 Public Health Ontario, Toronto, ON M5G 1M1, Canada; 8 Institute of Health Policy, Management, and Evaluation, University of Toronto, Toronto, ON M5T 3M6, Canada

**Keywords:** hepatitis C elimination, economic model, cost–utility analysis

## Abstract

Background: The World Health Organization (WHO) has set hepatitis C (HCV) elimination targets for 2030. Understanding existing gaps in the “HCV care-cascade” is essential for meeting these targets. We aimed to identify the level of service scale-up needed along the “HCV care-cascade” to achieve the WHO’s HCV elimination targets in Ontario, Canada. Methods: By employing a decision analytic model, we projected the quality-adjusted life years (QALYs) and healthcare costs for individuals with HCV in Ontario. We increased RNA testing and treatment rates to 98%, followed by increasing antibody testing uptake until we achieved the WHO’s mortality target (i.e., a 65% reduction in liver-related mortality by 2030 vs. 2015). Results: Without scaling up by 2030, the expected QALYs and costs per person were 9.156 and CAD 48,996, respectively. Improved RNA testing and treatment rates reduced liver-related deaths to 3.3/100,000, a 57% reduction from 2015. Further doubling the antibody testing rates can achieve the WHO’s mortality target in 2035, but not in 2030. Compared to the status quo, such program would be cost-effective considering a 50,000 CAD/QALY gained threshold if annual implementation costs stayed under 2.3 M CAD/100,000 people. Conclusions: Doubling the antibody testing rates, along with increased RNA testing and treatment rates, showed promise in meeting the WHO’s goals by 2035.

## 1. Introduction

Chronic hepatitis C (CHC) affects more than 55 million individuals worldwide and is known to cause fatal complications, such as liver failure and hepatocellular carcinoma (HCC). It is estimated that liver disease induced by the hepatitis C virus (HCV) accounts for approximately 290,000 deaths annually worldwide [1]. 

The introduction of highly effective direct-acting antivirals (DAAs) in 2013 revolutionized CHC management. These drugs demonstrated over 90% cure rates and favorable safety profiles [2,3,4]. DAAs offer an opportunity to eliminate HCV as a public health concern. With the aim of reducing the burden of CHC, the World Health Organization (WHO) has set ambitious global elimination targets that entail a 90% reduction in disease incidence and a 65% reduction in associated mortality rates by 2030 relative to 2015 [5]. Specifically, the target is to reduce the number of infections globally from 6–10 million to 0.9 million and to decrease the number of deaths from 1.4 million to less than 500,000 by 2030 [5]. Furthermore, the WHO has set additional goals, aiming for a 90% diagnosis and an 80% treatment coverage by 2030. 

Canada, similar to other developed nations, has committed to attain these goals [6,7]. Planning for HCV elimination, however, requires an accurate understanding of the existing gaps along the “HCV care-cascade”, including diagnosis, linkage to care, treatment, and cure. Identifying these gaps allows us to estimate the extent and focus of the scale-up necessary to achieve the elimination targets.

Canada adopted risk-based screening of individuals who are at a higher risk of HCV acquisition that involve any potential exposure to contaminated blood or products, e.g., patients who inject drugs, undergoing hemodialysis, or receiving blood transfusions [8]. Diagnosis normally involves two steps: first, a blood sample for antibody testing, and if the result is positive, a second blood sample for RNA confirmation. Multiple appointments pose challenges for patients and decrease testing rates, particularly among a difficult-to-reach population, e.g., immigrants, people who inject drugs, and people who are homeless. A recent population-based study in Ontario reported that out of 108,428 individuals carrying HCV antibodies, only 88% underwent RNA confirmation testing [9]. Among those who tested RNA positive, only 53% initiated treatment. This study also found that older birth cohorts, long-term residents, individuals with a history of substance use disorder, and social marginalization demonstrated lower rates of engagement with almost every step of HCV care [9].

The introduction of a simplified testing procedure (e.g., reflex RNA testing or dried blood spot testing in those with a positive antibody test using the blood collected at a single visit) has the potential to reduce the loss to care between the antibody and RNA testing. The purpose of our study is to identify the optimal level of service scale-up and investments needed along the “HCV care-cascade” to meet the WHO’s elimination targets by 2030 in Ontario, Canada’s most populous province, using a modeling-based approach.

## 2. Methods

### 2.1. Population

The modeled population is representative of individuals living with hepatitis C in Ontario as of 1 January 2019, informed by linked population-level health and administrative data held at the ICES (formerly known as the Institute of Clinical Evaluative Sciences) [9]. We stratified by three age cohorts: 5% of individuals with CHC were born before 1945, 49% were born between 1945 and 1965, and 46% were born after 1965, with corresponding mean ages of 78, 58, and 39 years old, respectively [9,10]. The majority of individuals (60%) were male [9]. The baseline characteristics of the modeled population are displayed in Table 1.

### 2.2. Strategies

We compared strategies aiming to increase CHC diagnosis and treatment uptake in Ontario to achieve the WHO target of a 65% reduction in liver-related mortality by 2030, relative to 2015, when 6.9 deaths per 100,000 people were reported [12]. We assumed that only individuals with HCC, decompensated cirrhosis (DC), and liver transplant were at risk of liver-related mortality.

We considered the following three strategies:

(i) A “status quo” strategy, which refers to the existing two-step diagnostic process with current diagnosis and treatment coverage. The annual probability of receiving an HCV antibody test was estimated using the back-calculation model and ranges from 0.040 to 0.127 depending on a birth cohort (Table S1) [10]. Of those who tested positive on the antibody test, 88% received the confirmatory RNA test, and of those who tested RNA positive, 53% received an antiviral treatment, mirroring the current care cascade [9]. Individuals not receiving RNA tests (12%) and not initiating treatment (47%) were assumed to be lost to care.
(ii) An “improving linkage to care” strategy. This strategy considers reflex testing that increases the proportion of RNA testing to 98% and treatment uptake to 98% for individuals who tested RNA positive. The near-optimal rates for RNA testing and treatment uptake were chosen considering feasibility and constraints in real-world healthcare settings.
(iii) “Reaching the undiagnosed population” by scaling up antibody testing in addition to the measures described under the second strategy to achieve a 65% reduction in liver mortality in 2030 as per the WHO’s mortality target. We assumed one-time screening. Considering that screening rates in the US surged by 50% following recommendations for birth cohort screening [13], we allowed for a potential 100% increase in the current antibody testing rates as the maximum limit, since exceeding this might not be feasible.

### 2.3. Model Structure and Assumptions

**Figure 1:**
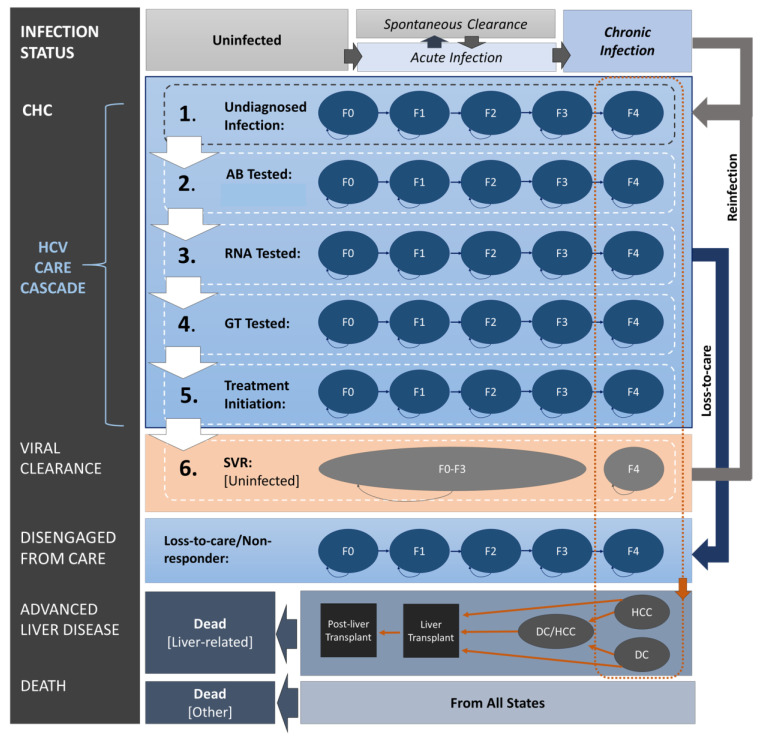
Model structure. Figure shows (i) HCV infection status (uninfected or infected with acute and chronic infection), (ii) HCV care cascade (undiagnosed, AB, RNA, genotype tested, treated, or SVR attained), (iii) disengagement from care, and (iv) natural progression of chronic hepatitis C (fibrosis stages (F0–F4), DC, HCC, liver transplantation, and death). Arrows display allowed transitions between health states. Transitions to treatment initiation and SVR status for individuals with HCC and/or DC are not illustrated. AB: antibody; CHC: chronic hepatitis C; DC: decompensated cirrhosis; F0–F4: fibrosis stages, where F4 is cirrhosis; GT: genotype; HCC: hepatocellular carcinoma; RNA: ribonucleic acid; SVR: sustained virologic response; HCV: hepatitis C virus.

We developed a state transition model using TreeAgePro 2020 (TreeAge Software Inc., Williamstown, MA, USA) to project the health and economic outcomes associated with improving the HCV care cascade in Ontario. The model is based on previous published CHC policy models [14,15,16] and comprises health states representing (i) the HCV infection status (i.e., uninfected, spontaneous clearance, and CHC), (ii) the HCV care cascade (e.g., undiagnosed infection, antibody tested, RNA tested, genotype tested, treatment initiated, and sustained virologic response (SVR) achieved), (iii) disengagement from care (e.g., not receiving confirmatory testing, not initiating treatment, discontinuing HCV treatment, or being a non-responder), and (iv) the natural history of CHC (i.e., fibrosis stages (F0–F4), DC, HCC, liver transplant, post-liver transplant, liver-related mortality, and reinfection). A structured diagram with modeled health states and the transitions between them is displayed in Figure 1. 

The hypothetical cohort entered the model based on the initial health state membership at baseline (Table 1). The cohort then progressed both in terms of disease history and across the HCV care cascade in weekly time steps (cycle length) until death. 

We assumed that uninfected individuals under the age of 50 years could acquire an HCV infection or remain uninfected. All individuals with new infections could either spontaneously clear the virus or progress through CHC health states (F0–F4) until reaching advanced liver disease. Those at an advanced liver disease stage (HCC and/or DC) could receive a liver transplant and enter a post-transplant state. Individuals with HCC, DC, and liver transplants were assumed to be at risk of liver-related mortality.

Both uninfected individuals and those with undiagnosed infection were eligible for one-time antibody testing at any point in time. We considered increased testing rates for the third strategy, “reaching the undiagnosed population”. Individuals with acute infection had a positive antibody test followed by a negative RNA test. Individuals with an undiagnosed CHC could be diagnosed and either continue across the HCV care cascade or become disengaged from care. Upon obtaining a positive antibody test result, RNA and genotype testing followed. In the status quo scenario, 88% of those who tested positive on an antibody test underwent a confirmatory RNA test [9], and in the comparator strategies, the rates were calibrated up to 98% to reflect the improving linkage-to-care-approach. 

Upon an HCV diagnosis, individuals may receive antiviral treatment within an assumed 6-month timeframe. In the status quo scenario, 53% of those diagnosed with HCV initiated treatment [9], while under the two comparator strategies, the treatment rate was calibrated up to 98%. The fibrosis progression stopped while the individuals were on treatment. Individuals who did not respond to the first-line treatment (sofosbuvir/velpatasvir) could initiate a second-line treatment (sofosbuvir/velpatasvir/voxilaprevir). The SVR for both lines of treatment were assumed to be the same but overall lower for patients with cirrhosis and DC [3,4,17]. Patients who failed the second-line treatment were classified as non-responders and assumed to not be retreated thereafter. Those who did not receive confirmatory testing after a positive antibody test, those who did not initiate treatment, and those who discontinued or did not respond to treatment were considered to be disengaged from care and continued the progression through fibrosis stages.

We assumed that patients who achieved an SVR at earlier stages of fibrosis (F0–F3) would no longer progress, whereas those who achieved an SVR following the development of cirrhosis (F4) could progress to advanced liver disease (i.e., DC or HCC) but at a reduced rate [18]. Similarly, individuals with advanced liver disease (HCC and/or DC) who attained SVR had a lower mortality hazard compared to the untreated individuals. 

Furthermore, individuals who achieved an SVR may become re-infected. The reinfection rate was derived from a systematic review based on rates reported for low-risk populations [19]. Re-infected individuals would re-enter the care cascade as undiagnosed at the same fibrosis stage (F0-F4) as that at the time of the SVR; upon re-infection, they would become eligible for HCV antibody and RNA testing and subsequent antiviral treatment, maintaining identical assumptions and probabilities for HCV testing, antiviral treatment, and SVR rates as those for primary-infected individuals.

### 2.4. Health and Economic Outcomes

We conducted this evaluation from the Canadian provincial health system perspective, considering a 12-year program time horizon (until the end of 2030) and lifetime individual time horizon. Our primary outcomes were quality-adjusted life years (QALYs) and direct health system costs (in 2023 CAD) accumulated over 12 years, associated with the level of scale-up in health services that would result in a 65% reduction in liver mortality in 2030 relative to 2015. The QALYs and costs were discounted at 1.5% annually as per the Canadian economic evaluation guidelines [20]. Secondary outcomes were CHC diagnosis and treatment rates by 2030 and expected life years (LYs). We also considered a longer time horizon to determine when WHO targets would be reached if not by 2030.

### 2.5. Data

The prevalence of CHC, the proportion of undiagnosed cases, their fibrosis stages (F0–F4), and the probability of HCV antibody testing were collected from back-calculation modeling [11]. The distribution of fibrosis stage among individuals diagnosed with CHC, as well as their placement within the HCV care cascade, was obtained from a population-based study in Ontario [9] (Table 1 and Table S1). 

For individuals infected with HCV, the disease progression was based on the natural history of CHC as indicated by stage-specific fibrosis progression rates [21]. We used Canadian lifetables to estimate mortality by age and sex [22]. All other clinical probabilities, such as SVR and adverse event rates, were collected from recently published studies [4,18,23,24,25] (Table S1).

Utility values for CHC health states were obtained from a published meta-regression capturing over 18,000 utility measurements [26]. Utility values were standardized to the EuroQol-5D-5L instrument. The meta-regression model included age, sex, liver disease stage, treatment, and SVR status. The results show substantial utility decrements associated with cirrhosis, DC, and HCC; a small utility decrement during active DAA therapy; and a small improvement in utility for individuals who achieve an SVR. These estimates were incorporated into our decision analytic model (Table S2). Individuals without CHC were considered to have the same utility as the general Canadian population [27].

Health system costs from the public payer perspective were obtained from a recent population-based retrospective cohort study using administrative data in Ontario [28]. The study included over 48,000 individuals aged 18-105 years diagnosed with CHC during the 2003–2014 period. The authors estimated direct medical costs (in 2018 CAD) based on disease severity. The study segmented the observation time of individuals from diagnosis until death or the end of follow-up into stages based on disease severity and estimated costs of being uncured during each of these following stages: no cirrhosis, cirrhosis, DC, HCC, DC and HCC, liver transplantation, and terminal phases (6 months prior to death) for individuals with and without advanced liver disease. These estimates, adjusted to 2023 CAD using the consumer price index [29], were incorporated into our decision analytic model (Table S3). 

### 2.6. Analysis

The WHO aims to achieve a 65% reduction in liver-related mortality by 2030 compared to the 2015 levels [5]. In 2015, the liver-related mortality rates were 6.9/100,000 in Canada [12]. The WHO’s target of a 65% reduction implies achieving a mortality rate of 2.4/100,000 by 2030. The extent of scale-up needed along the HCV care cascade to achieve this mortality target was incorporated in two steps. First, we implemented the strategy with increased RNA testing (98%) and treatment uptake (98%) rates aimed to determine the extent to which this approach could achieve a 65% reduction in liver-related mortality relative to 2015 levels. The calibration process involved systematically increasing RNA testing (from 88% to a maximum of 98%) and treatment rates (from 53% to a maximum of 98%) until either approaching the target or reaching the maximum limit, whichever occurred first. If the target was not achieved through the maximum increase in RNA and treatment (up to 98% each), we then scaled up antibody testing following the same process. We allowed for a potential two-fold increase in the current antibody testing rates as a theoretical maximum. Additionally, we determined the expected year for reaching the mortality target if it was not achieved by 2030. Calibration was carried out through deterministic calculations using bound optimization by quadratic approximation algorithm (BOBYQA) [30]. The goodness-of-fit assessment was based on the simple sum of squared differences. 

We performed a probabilistic sensitivity analysis (PSA), where the key parameters (fibrosis progression, CHC-related costs, and utilities) were randomly sampled from their respective distributions, with means based on deterministic base-case values and variances from 95% confidence intervals (CIs) summarized in Tables S1–S3.

Policy and decision makers often use cost-effectiveness thresholds to guide adoption and funding decisions for health services. Though there is no single threshold recommended for Canada, CAD 50,000 per QALY is a commonly accepted threshold [31]. In the base-case analysis, we did not account for the program implementation costs associated with these strategies. Anticipated operating expenses for an agency dedicated to enhancing linkage to care or antibody testing could include staff salaries, outreach initiatives, operational space, computer hardware and software, and other expenses. Instead, we adopted a “reverse” cost-effectiveness analysis approach to support programming decisions, estimating the maximum cost the program could incur while remaining cost-effective at a CAD 50,000 per QALY gained threshold. 

## 3. Results

### 3.1. Meeting WHO Targets

Under the status quo, we projected a total of 1313 individuals with CHC per 100,000 people between 2019 and 2030 in Ontario. Of those, 946 per 100,000 individuals would be diagnosed (72%) at F0–F4 stages before developing end-stage liver disease (ESLD), such as DC and HCC; 567 per 100,000 (43%) would receive antiviral treatment; and 546 per 100,000 (42%) would achieve an SVR. We estimated that 83/100,000 individuals would develop ESLD by 2030 in addition to 47/100,000 individuals with existing ESLD from 2019. Between 2019 and 2030, we estimated that 81/100,000 individuals would die due to liver-related complications, with 6.7/100,000 deaths in 2030 alone (Table 2). 

The second strategy aimed at improving linkage to care, with 98% RNA testing and 98% treatment initiation rates among individuals who tested positive, resulting in 77% of individuals with CHC being diagnosed (the WHO’s target is 90%) and 75% (the WHO’s target is 80%) being treated (Table 2). This corresponded to a reduction of 37/100,000 individuals with ESLD and a reduction of 21/100,000 liver deaths by 2030 relative to the status quo. We estimated 3.4/100,000 liver-related deaths in 2030, marking a 51% reduction relative to the rate in 2015, which was 6.9 deaths per 100,000 individuals (the WHO’s target is 65% reduction). 

Doubling the current annual antibody testing rate in addition to increased RNA testing and treatment coverage in the third strategy, “reaching the undiagnosed population”, resulted in 85% of individuals with CHC being diagnosed and 83% being treated (Table 2). Liver-related mortality decreased by 57% relative to 2015, reaching 2.95 per 100,000 in 2030, slightly below the WHO’s mortality target. 

Extending the time horizon to 2035 would enable Canada to achieve the WHO’s mortality target using the third strategy (Table 2). A twofold increase in the current annual HCV antibody testing rate, along with increased RNA testing and treatment rates, would allow for 86% and 84% of individuals with CHC to be diagnosed and treated, respectively. Between 2019 and 2035, we estimated that 47/100,000 liver-related deaths will be prevented compared to the status quo. In 2035 alone, the estimated liver-related mortality rate would be 2.41/100,000, reaching WHO’s 65% reduction target relative to 2015. The PSA showed that this target was attained in 52% of simulations, with a mean rate of 2.41 (95%CI: 2.15–2.69)/100,000. The results by birth cohorts are presented in Tables S4–S6. 

### 3.2. Cost-Effectiveness

Under the status quo, the total accumulated QALYs and costs by 2030 were estimated to be 9.155 and CAD 56,791 per person, respectively. Relative to the status quo, the strategy aimed at “improving linkage to care” saved 0.002 QALYs per person. Excluding program implementation costs, it cost CAD 1018 per QALY gained compared to the status quo (Table 2). For this strategy to remain cost-effective at a threshold of 50,000 CAD/QALY, the annual program implementation cost per 100,000 people can be up to CAD 852,000. Extending the time horizon to 2035 resulted in greater cost savings and QALY gains due to the prevention of ESLD and liver-related deaths. Consequently, the annual program implementation cost per 100,000 people could be CAD 2,049,000 (Table 2).

The “improving linkage to care” strategy was cost-effective at a threshold of 50,000 CAD/QALY for the entire cohort and among the three birth cohorts when analyzed separately (Tables S4–S6). For this strategy to remain cost-effective at a threshold of 50,000 CAD/QALY, the annual program implementation cost per 100,000 people may be CAD 702,000, CAD 2,083,000, and CAD 450,000, for the <1945, 1945–1965, and >1965 birth cohorts, respectively, for the 2030 time horizon. The implementation costs could be CAD 1,543,000, CAD 3,755,000, and CAD 1,072,000 for the <1945, 1945–1965, and >1965 birth cohorts, respectively, for the 2035 time horizon. The PSA showed that “improving linkage to care” remains cost-effective under these calculated thresholds in >52% of simulations for all three birth cohorts for the 2035 horizon.

A further increase in the antibody testing rate for the third strategy, “reaching the undiagnosed population”, saved 0.0004 QALYs per person by 2030 and cost (excluding the program cost) CAD 52,505 per QALY gained relative to the “improving linkage to care” strategy. The strategy was cost-effective only for the 1945–1965 birth cohort for the 2030 time horizon, and for the 1945–1965 and >1965 birth cohorts for the 2035 time horizon. For this strategy to remain cost-effective at a threshold of 50,000 CAD/QALY, the annual program implementation cost per 100,000 people may reach up to CAD 123,000 for the 1945–1965 birth cohort for the 2030 time horizon and up to CAD 335,000 and CAD 288,000 for the 1945–1965 and >1965 birth cohorts, respectively, for the 2035 time horizon (Tables S4–S6). The PSA showed that the “reaching the undiagnosed population” approach remains cost-effective under these calculated thresholds in >51% of simulations for the 1945–1965 and >1965 birth cohorts for the 2035 time horizon.

## 4. Discussion

Our study aimed to assess the health and economic implications of scaling up the HCV care cascade in Ontario, Canada to achieve the WHO’s ambitious target of a 65% reduction in liver-related mortality by 2030 using a modeling-based approach. Our results suggest that while significant progress can be made to meet the WHO’s diagnosis and treatment targets, meeting the mortality target by 2030 appears challenging. Improvement in the linkage to care achieved a 51% reduction in liver deaths by 2030. Doubling the antibody testing rate alongside RNA testing and treatment coverage (“reaching undiagnosed population”) further reduced liver-related mortality by 57%, which is below the WHO’s target. Only when the time horizon was extended to 2035 did we achieve a 65% reduction in liver-related mortality under the “reaching the undiagnosed population” strategy. Although this progress would be substantial, it emphasizes the complexity of achieving such ambitious goals within the given timeframe.

The analysis highlights that without intervention, diagnosis and treatment rates would fall behind the WHO’s targets, projected at 72% and 43%, respectively, with no observable reduction in mortality. However, even maintaining current rates presents challenges, particularly in light of the COVID-19-related disruptions to health systems. During the first wave of the pandemic in Ontario, HCV testing decreased drastically, only recovering to 72% of the pre-pandemic levels by 2021 in Ontario [32] and to the near pre-restriction levels in British Columbia [33]. The DAA dispensing rate also declined by close to 50% compared to the pre-pandemic era in Ontario and by 26% in British Columbia, but it was recovered by 2022 [34,35].

Adopting strategies to improve linkage to care is imperative for Canada to ensure progress towards HCV elimination efforts. As shown, a strategy with enhanced RNA testing and treatment rates would bring diagnosis and treatment levels closer to the WHO’s targets at 78% and 76%, respectively, achieving a 51% reduction in mortality relative to the 2015 year. 

Setting the limits for RNA testing and treatment uptake at 98% each was deemed feasible, considering the current RNA testing rate of 88% [9] and expert opinion suggesting a current treatment rate of 95% [10]. We showed that for this strategy to remain cost-effective at a 50,000 CAD/QALY threshold, the annual program implementation cost per 100,000 people could reach up to CAD 850 K for the entire cohort and over 2 M CAD/100,000 people for the 1945–1965 birth cohort (Table 2 and Table S5). The ability to allocate higher resources in the latter cohort was attributed to cost savings and QALY gains resulting from prevented ESLD cases, which were more pronounced in this cohort. 

Yet, achieving the WHO mortality target required doubling the current screening rates and extending the time horizon to 2035. Only under the extended time horizon did the “reaching the undiagnosed population” strategy become cost-effective, albeit for the 1945–1965 and >1965 birth cohorts only. To remain cost-effective compared to the “improving linkage to care” strategy, the annual program implementation cost per 100,000 people for the “reaching the undiagnosed population” strategy may reach CAD 288 K and CAD 335 K for the 1945–1965 and >1965 birth cohorts, respectively. With the >1965 birth cohort size in Ontario being close to 10 million, this would translate to an operational cost of CAD 29 M. However, the anticipated cost for operating the program is expected to be notably lower, especially when considering that Ontario allocated approximately CAD 34 M toward colorectal, breast, and cervical cancer screening programs (combined) in the 2019–2020 period [36]. 

Our results align with the recently published model by Feld et al., where authors examined Canada’s progress towards HCV elimination [37]. They reported that certain provinces, including Ontario, are falling short of meeting elimination targets by 2030 if current trends persist, highlighting the need for substantial increases in annual treatments to meet these targets. Evidence on the cost-effectiveness of point-of-care testing strategies for HCV care is also evolving. Shih et al. evaluated the cost-effectiveness of point-of-care testing strategies compared to standard-of-care testing in various settings [38]. They found that combined point-of-care HCV antibody and reflex point-of-care RNA testing are cost-effective strategies in settings with high- risk populations.

Canada is still in the process of implementing strategies to reduce the number of steps in the care cascade to achieve hepatitis C virus (HCV) elimination. Currently, eight Canadian provinces, all except for Ontario and Quebec, have implemented reflex testing using hepatitis C RNA tests to ensure that those who are antibody positive also receive an RNA test. Most the provinces are piloting dried blood spot testing, while Ontario has already implemented it [39]. To date, only British Columbia has adopted birth-cohort-based screening [40].

While our study provides valuable insights into HCV elimination in Canada, we acknowledge several limitations. Firstly, it is important to note that our study is based on a static cohort and does not account for immigration patterns in Canada, potentially underestimating projected individuals with CHC due migration from regions with a higher prevalence of HCV. On the other hand, our analysis did not evaluate the existing measures aimed at preventing the spread of HCV, such as harm reduction services, which may reduce CHC prevalence. Secondly, we incorporated just one-time screening; however, in populations at an increased risk of initial infection and reinfection, such as people who inject drugs, repeated testing might be needed. Lastly, while we acknowledged that the upper limit for the antibody testing rate might be challenging to achieve in a real-world setting, we also aimed to explore the potential scale-up required to meet the mortality target.

Our study contributes to the growing research landscape on HCV elimination targets by integrating information from systematic reviews, meta-analyses, and population-level health administrative records, leveraging a previously published and validated disease model. This is the first study that assessed the effects of mitigating loss-to-care on HCV elimination goals alongside the potential of further expanding screening services. Furthermore, we quantify the operational costs associated with the implementation of these HCV elimination strategies. As such, this study provided insights into both the clinical and the economic implications of HCV elimination efforts in Ontario.

## 5. Conclusions

HCV diagnosis and linkage to care remains a concern in Canada. Our study suggests that a strategy aimed to improve linkage to care could be cost-effective across all birth cohorts, particularly for the 1945–1965 birth cohort. Doubling the current antibody testing rates shows promise in achieving the WHO’s goals and could be cost-effective for the <1945 and 1945–1965 birth cohorts, especially when extending the timeframe to 2035. Interventions that streamline diagnosis and treatment into a single visit and, as such, address the gaps in the HCV care cascade should be prioritized.

## Figures and Tables

**Table 1:** Baseline characteristics of cohort (as of 31 December 2018).

	>1965	1965–1945	>1945	Source
Uninfected individuals (%)	99.26	97.62	99.08	[10,11] ^†^
Acute hepatitis C (%)	0.11	0.64	0.16	[10,11] ^††^
Chronic hepatitis C (CHC) (%)	0.63	1.74	0.76	[11]
Proportion of undiagnosed CHC (%)	42.39	15.72	19.71	[11]
No cirrhosis (%)	80.9	50.2	50.2	[10]
Cirrhosis (%)	19.1	49.8	49.8	[10]
Proportion diagnosed CHC (%)	57.61	84.28	80.29	[10]
Age	39	58	78	[9,10]
Male (%)	57.0	65.0	46.0	[9]
No cirrhosis (%)	93.8	77.5	76.0	[9]
Cirrhosis (%)	4.2	12.8	12.0	[9]
DC (%)	1.8	6.6	7.2	[9]
HCC (%)	0.2	3.1	4.8	[9]
Proportion with RNA positive test (%)	48.1	61.5	50.4	[9]
Proportion with viral genotype on record (%)	44.1	58.9	47.6	[9]
Proportion initiated antiviral therapy (%)	21.7	36.6	25.8	[9]
Proportion achieved SVR (%)	15.4	28.8	19.4	[9]

CHC: chronic hepatitis C; DC: decompensated cirrhosis; HCC: hepatocellular carcinoma; RNA: ribonucleic acid; SVR: sustained virologic response. ^†^ Computed as a complement, e.g., for the >1965 cohort: 100 − 0.11 − 0.63 = 99.26. ^††^ Defined as individuals who had a positive antibody test but a negative RNA test, and no record of antiviral treatment.

**Table 2:** The CHC care cascade and health and economic outcomes over the 2019-2030 and 2019-2035 periods for the entire cohort.

		Predicted Cases as of 31 December 2030 ^1^ per 100,000 People			Predicted Cases as of 31 December 2035 ^1^ per 100,000 People		
	Estimated Cases as of January 2019 ^2^	Status Quo	RNA Rate = 98% and Tx Rate = 98%	RNA Rate = 98% and Tx Rate = 98% AB Testing Rate x2	Status Quo	RNA Rate = 98% and Tx Rate = 98%	RNA Rate = 98% and Tx Rate = 98% and AB Testing Rate x2
**Care cascade outcomes**							
Total CHC cases ^3^	986	1313 (100%)	1313 (100%)	1313 (100%)	1448 (100%)	1448 (100%)	1448 (100%)
Diagnosed at F0–F4 stages ^4^	383	946 (72%)	1012 (77%)	1124 (85%)	1060 (73%)	1140 (78%)	1258 (86%)
Initiated antiviral therapy ^5^	203	567 (43%)	982 (75%)	1190 (83%)	623 (43%)	1106 (76%)	1221 (84%)
Achieved SVR ^5^	153	546 (42%)	940 (71%)	1044 (79%)	600 (41%)	1060 (73%)	1144 (80%)
ESLD ^6^	47	130 (10%)	93 (7%)	86 (6%)	169 (12%)	107 (8%)	97 (7%)
**Health and economic outcomes**							
Liver-related mortality, cumulative	NA	81	60	56	116	75	69
Liver-related mortality in 2030, 2035	NA	6.73	3.43	2.95	7.07	2.86	2.41
Life years per person	NA	11.583	11.584	11.584	16.076	16.078	16.079
QALYs per person	NA	9.155	9.157	9.157	12.239	12.242	12.243
Costs per person ^7^	NA	CAD 56,791	CAD 56,793	CAD 56,812	CAD 79,635	CAD 79,517	CAD 79,509
ΔQALY ^8^ per person	NA	-	0.0018	0.0004	-	0.0035	0.0007
ΔCost ^8^ per person	NA	-	CAD 2	CAD 19	-	CAD −118	CAD −7
ICUR ^8^ (sequential)	NA	-	**CAD 1018**	**CAD 52,505**	-	**Cost saving**	**Cost saving**
**Program implementation’s maximum cost per 100,000 people for the strategy to remain cost-effective at 50,000 CAD/QALY gained threshold**							
Compared to the “Status quo”	NA	ref.	**CAD 852,273**	-	ref.	**CAD 2,049,381**	**CAD 2,329,160**
Compared to the previous strategy	NA	-	-	-	-	ref.	**CAD 279,779**

AB: antibody; CAD: Canadian dollar; CHC: chronic hepatitis C; ESLD: end-stage liver disease; F0–F4: fibrosis stages, where F4 is cirrhosis; ICUR: incremental cost/utility ratio; QALY: quality-adjusted life years; ref: reference; RNA: ribonucleic acid; SVR: sustained viral response; Tx: treatment. ^1^ Cumulative number of cases over 2019–2030 period and 2019–2035 period, including those who were alive as of 1 January 2019 (i.e., at beginning of simulation). Percentages were calculated out of total CHC cases. ^2^ Number of cases at beginning of simulation. ^3^ Total CHC cases included diagnosed and undiagnosed F0–F4 cases, and those with ESLD. ^4^ Diagnosed, i.e., tested RNA positive, including cases before developing ESLD. ^5^ Individuals who initiated therapy and/or achieved SVR before developing ESLD. ^6^ End-stage liver disease including decompensated cirrhosis, hepatocellular carcinoma, and liver transplantation. ^7^ Costs represent total healthcare cost, excluding program implementation cost, rounded to nearest dollar and expressed in 2023 Canadian dollars. ^8^ ΔQALY, ΔCost (incremental values) and ICUR were calculated relative to prior less costly strategy and excludes program implementation-related costs.

## Data Availability

All data generated or analyzed during this study are included in this published article (and Supplementary Materials).

## Supplementary Materials

The following supporting information can be downloaded at: https://www.mdpi.com/article/10.3390/v16081224/s1, Table S1: Clinical parameters. Table S2: Utilities. Table S3: Costs in 2023 CAD. Table S4: CHC care cascade and health and economic outcomes over 2019–2030 and 2019–2035 periods for <1945 birth cohort. Table S5: CHC care cascade and health and economic outcomes over 2019–2030 and 2019–2035 periods for 1945–1965 birth cohort. Table S6: CHC care cascade and health and economic outcomes over 2019–2030 and 2019–2035 periods for >1965 birth cohort.
