# Supplementary material for: Bridging Hepatitis C Care Gaps: A Modeling Approach for Achieving the WHO’s Targets in Ontario, Canada
Source: Viruses. 2024 Jul 31;16(8):1224. doi: 10.3390/v16081224 (PMC11359558; doi:10.3390/v16081224)
Supplement: Supplementary file 1 [file viruses-16-01224-s001.zip › viruses-3114287-supplementary.pdf]

Supplemental Material

Supplemental Table S1. Clinical Parameters 2
Supplemental Table S2. Utilities 4
Supplemental Table S3. Costs in 2023 CAD 5
Supplemental Table S4. CHC care cascade and health and economic outcomes over 2019-2030 and 2019-2035 periods for <1945 birth cohort 6
Supplemental Table S5. CHC care cascade and health and economic outcomes over 2019-2030 and 2019-2035 periods for 1945-1965 birth cohort 7
Supplemental Table S6. CHC care cascade and health and economic outcomes over 2019-2030 and 2019-2035 periods for >1965 birth cohort 8
References 9

Supplemental Table S1. Clinical Parameters

	Mean	Lower value	Upper value	Source
**Hepatitis C infection**				
Incidence (for age 18-50)	0.00054	-	-	[1]
Reinfection post-SVR for low-risk population (per 1,000 person-years)	1.850	0.710	3.350	[2]
Spontaneous clearance	0.260	0.220	0.290	[3]
**Chronic hepatitis C progression**				
Annual probability of progressing from F0 to F1	0.107	0.097	0.118	[4]
Annual probability of progressing from F1 to F2	0.082	0.074	0.091	[4]
Annual probability of progressing from F2 to F3	0.117	0.107	0.129	[4]
Annual probability of progressing from F3 to F4	0.116	0.104	0.131	[4]
Annual probability of progressing from F4 to DC (non-SVR)	0.036	0.027	0.043	[5]
Annual probability of progressing from F4 to HCC (non-SVR)	0.024	0.018	0.030	[5]
Annual probability of progressing from DC to HCC	0.060	0.011	0.080	[6]
Annual probability of liver transplant	0.033	0.026	0.038	[5]
Hazard ratio for risk of progression from F4 to HCC (SVR)	0.310	0.270	0.370	[7]
Hazard ratio for risk of progression from F4 to DC (SVR)	0.110	0.050	0.240	[7]
Annual probability of death from DC	0.216	0.162	0.270	[8]
Annual probability of death from HCC	0.380	0.310	0.510	[9]
Hazard ratio for risk of progression from DC/HCC to death (SVR)	0.250	0.220	0.300	[7]
Annual probability of death from liver transplant (1st year)	0.142	0.124	0.159	[10]
Annual probability of death from liver transplant (>1st year)	0.034	0.024	0.043	[10]
**Hepatitis C care cascade**				
Receive HCV antibody test for unknown CHC for those born <1945	0.040		calibrated	[11]
Receive HCV antibody test for unknown CHC for those born 1945-1965	0.090		calibrated	[11]
Receive HCV antibody test for unknown CHC for those born 1965	0.127		calibrated	[11]
Receive HCV antibody test for uninfected for those born <1945	0.0035		calibrated	[11]
Receive HCV antibody test for uninfected for those born 1945-1965	0.0082		calibrated	[11]
Receive HCV antibody test for uninfected for those born 1965	0.0046		calibrated	[11]

Receive HCV RNA test (if antibody test +)	0.880		calibrated	[12]
Initiate 1st line DAA treatment (if RNA+) (within 6 m of diagnosis)	0.530		calibrated	[12]
Probability of discontinuation (F0-F4) (SOF/VEL 12w)	0.005	0.001	0.010	[13, 14]
Initiate 2nd line DAA treatment	0.500	-	-	assumption
Probability of SVR-12 (F0-F3) (SOF/VEL or SOF/VEL/VOX 12w)	0.950	0.930	0.980	[15]
Probability of SVR-12 (F4) (SOF/VEL or SOF/VEL/VOX 12w)	0.890	0.820	0.930	[14, 16]
Probability of SVR-12 decompensated cirrhosis	0.860	0.830	0.880	[17]
**Hepatitis C testing**				
Sensitivity of antibody test	0.980	0.950	1.000	[18]
Specificity of antibody test	1.000	0.950	1.000	[18]

DAA: direct-acting antivirals; CHC: chronic hepatitis C; DC: decompensated cirrhosis; F0-F4: fibrosis stages, where F4 is cirrhosis; HCC: hepatocellular carcinoma; HCV: hepatitis C virus; RNA: ribonucleic acid; SOF/VEL: sofosbuvir/ velpatasvir; SOF/VEL/VOX: sofosbuvir/ velpatasvir/voxilaprevir; SVR: sustained virologic response.

Supplemental Table S2. Utilities

**Utilities for general population**	**Mean**	**SE**	**Source**
Age 18-24	0.879	0.102	[19]
Age 25-34	0.881	0.122	[19]
Age 35-44	0.878	0.094	[19]
Age 45-54	0.855	0.130	[19]
Age 55-64	0.839	0.140	[19]
Age 65-74	0.867	0.113	[19]
Age 74+	0.861	0.109	[19]
**Utilities for CHC**	**Mean**	**95% CI**	
No cirrhosis [F0-F3]	0.806	0.767 – 0.845	[20]
Compensated cirrhosis [F4]	0.726	0.680 – 0.772	[20]
DC	0.657	0.602 – 0.711	[20]
HCC	0.717	0.647 – 0.788	[20]
Post-transplantation	0.712	0.657 – 0.767	[20]
SVR post-treatment	0.841	0.801 – 0.880	[20]
Disutility of being on DAA therapy	-0.019	0.006 – 0.031	[20]

DAA: direct-acting antivirals; CI: confidence interval; CHC: chronic hepatitis C; DC: decompensated cirrhosis; HCC: hepatocellular carcinoma; SE: standard error; SVR: sustained virologic response.

Supplemental Table S3. Costs in 2023 CAD

Costs	Mean	95% CI	Source
Cost of SOF/VEL (12 week)	60,000	45,000 – 75,000	[21]
Cost of SOF/VEL/VOX (12 week)	60,000	45,000 – 75,000	[21]
**Costs for diagnosis**			
Cost of personnel per test	28	20 - 32	[22]
Cost of antibody test	28	21 - 35	[22]
Cost of RNA test	133	100 - 167	[22]
**30-day cost for CHC**			
No cirrhosis [F0-F3]	1,201	1,144 – 1,256	[23]
Compensated cirrhosis [F4]	3,164	2,820 – 3,508	[23]
DC	4,970	4,525 – 5,416	[23]
HCC	5,408	3,934- 6,881	[23]
DC+HCC	9,423	7,451 – 11,395	[23]
Liver Transplant	7,572	5,409 – 9,734	[23]
SVR	853	802 - 906	[23]
Liver-related death (last 6 months of life)	12,113	11,314 – 12,913	[23]
Non-liver related death (last 6 months of life)	9,017	8,316 – 9,719	[23]
**Annual cost of care for uninfected individuals, by age**			
Age 15-25	2,023	1,963 – 2,084	[22, 24]
Age 26-35	1,984	1,944 – 2,023	[22, 24]
Age 36-45	2,203	2,159 – 2,248	[22, 24]
Age 46-55	2,870	2,842 – 2,900	[22, 24]
Age 56-65	4,769	4,629 – 4,913	[22, 24]
Age 66-75	7,390	7,244 – 7,540	[22, 24]
Age 76+	9,041	8,685 – 9,409	[22, 24]

CAD: Canadian dollar; CI: confidence interval; CHC: chronic hepatitis C; DC: decompensated cirrhosis; HCC: hepatocellular carcinoma; RNA: ribonucleic acid; SOF/VEL: sofosbuvir/ velpatasvir; SOF/VEL/VOX: sofosbuvir/ velpatasvir/voxilaprevir; SVR: sustained viral response

Supplemental Table S4. CHC care cascade and health and economic outcomes over 2019-2030 and 2019-2035 periods for <1945 birth cohort

	Predicted cases as of Dec 31, 2030[1] per 100,000 people			Predicted cases as of Dec 31, 2035[1] per 100,000 people		
	**Status quo**	**RNA rate = 98% and Tx rate=98%**	**RNA rate = 98% and Tx rate = 98% and AB testing rate x2**	**Status quo**	**RNA rate = 98% and Tx rate = 98%**	**RNA rate = 98% and Tx rate = 98% and AB testing rate x2**
**Care cascade outcomes**						
Total CHC cases [2]	660 (100%)	660 (100%)	660 (100%)	660 (100%)	660 (100%)	660 (100%)
Diagnosed at F0-F4 stages [3]	467 (71%)	494 (75%)	516 (78%)	470 (71%)	496 (75%)	519 (79%)
Initiated antiviral therapy [4]	290 (44%)	480 (73%)	501 (76%)	291 (44%)	482 (73%)	504 (76%)
Achieved SVR [4]	279 (42%)	458 (69%)	478 (72%)	280 (42%)	461 (70%)	481 (73%)
ESLD [5]	126 (19%)	102 (16%)	99 (15%)	136 (21%)	106 (16%)	103 (16%)
**Health and economic outcomes**						
Liver-related mortality, cumulative	74	61	59	85	66	65
Liver-related mortality in 2030, 2035	3.33	1.95	1.79	1.38	0.69	0.62
Life years per person	8.865	8.866	8.866	10.323	10.324	10.324
QALYs per person	6.314	6.315	6.315	7.147	7.148	7.149
Costs per person [6]	CAD 204,041	CAD 204,028	CAD 204,035	CAD 249,874	CAD 249,835	CAD 249,839
ΔQALY [7] per person	-	0.0009	0.0001	-	0.0013	0.0001
ΔCost [7] per person	-	CAD −13	CAD 6	-	CAD −39	CAD 5
ICUR [7] (sequential)	-	**cost saving**	**CAD 118,069**	**-**	**cost saving**	**CAD 53,584**
**Program implementation's maximum cost for the strategy to remain cost-effective [8]**						
Cost per 100,000 people	-	**CAD 702, 519**	**-**	**-**	**CAD 1,072,415**	**-**

AB: antibody; CAD: Canadian dollar; CHC: chronic hepatitis C; ESLD: end-stage liver disease; F0-F4: fibrosis stages, where F4 is cirrhosis; ICUR: incremental cost/utility ratio; QALY: quality adjusted life years; RNA: ribonucleic acid; SVR: sustained viral response; Tx: treatment.

[1] Cumulative number of cases over 2019-2030 and 2019-2035 periods, including those who were alive as of January 01, 2019 (i.e., at beginning of simulation). Percentages were calculated out of total CHC cases.
[2] Total CHC cases included diagnosed and undiagnosed F0-F4 cases, and those with ESLD.
[3] Diagnosed, i.e., tested RNA positive, including cases before developing ESLD.
[4] Individuals who initiated therapy and/or achieved SVR before developing ESLD.
[5] End-stage liver disease including decompensated cirrhosis, hepatocellular carcinoma, and liver transplantation.
[6] Costs represent total healthcare cost, excluding program implementation cost, rounded to nearest dollar and expressed in 2023 Canadian dollars.
[7] ΔQALY, ΔCost (incremental values) and ICUR were calculated relative to prior less costly strategy and excludes program implementation-related costs.
[8] The strategy was considered cost-effective if the incremental costs relative to prior less costly strategy were CAD <50,000 per QALY gained.

Supplemental Table S5. CHC care cascade and health and economic outcomes over 2019-2030 and 2019-2035 periods for 1945-1965 birth cohort

	Predicted cases as of Dec 31, 2030[1] per 100,000 people			Predicted cases as of Dec 31, 2035[1] per 100,000 people		
	**Status quo**	**RNA rate = 98% and Tx rate=98%**	**RNA rate = 98% and Tx rate = 98% and AB testing rate x2**	**Status quo**	**RNA rate = 98% and Tx rate = 98%**	**RNA rate = 98% and Tx rate = 98% and AB testing rate x2**
**Care cascade outcomes**						
Total CHC cases [2]	1,785 (100%)	1,785 (100%)	1,785 (100%)	1,785 (100%)	1,785 (100%)	1,785 (100%)
Diagnosed at F0-F4 stages [3]	1,432 (80%)	1,500 (84%)	1,560 (87%)	1,448 (81%)	1,517 (85%)	1,569 (88%)
Initiated antiviral therapy [4]	956 (54%)	1,466 (82%)	1,525 (85%)	964 (54%)	1,483 (83%)	1,533 (86%)
Achieved SVR [4]	932 (52%)	1,420 (80%)	1,477 (83%)	940 (53%)	1,437 (81%)	1,486 (83%)
ESLD [5]	318 (18%)	245 (14%)	232 (13%)	385 (22%)	272 (15%)	255 (14%)
**Health and economic outcomes**						
Liver-related mortality, cumulative	208	164	157	275	198	188
Liver-related mortality in 2030, 2035	14.27	8.08	7.26	12.80	5.98	5.30
Life years per person	11.451	11.453	11.453	15.703	15.708	15.708
QALYs per person	9.019	9.022	9.023	11.972	11.977	11.978
Costs per person [6]	CAD 81,226	CAD 81,158	CAD 81,161	CAD 122,684	CAD 122,432	CAD 122,415
ΔQALY [7] per person	-	0.0030	0.0003	-	0.0055	0.0006
ΔCost [7] per person	-	CAD −68	CAD 2	-	CAD −252	CAD −17
ICUR [7] (sequential)	-	**cost saving**	CAD 7,810	-	**cost saving**	**cost saving**
**Program implementation's maximum cost for the strategy to remain cost-effective [8]**						
Cost per 100,000 people	-	**CAD 2,083,333**	**CAD 123,668**	-	**CAD 3,754,997**	**CAD 335,523**

AB: antibody; CAD: Canadian dollar; CHC: chronic hepatitis C; ESLD: end-stage liver disease; F0-F4: fibrosis stages, where F4 is cirrhosis; ICUR: incremental cost/utility ratio; QALY: quality adjusted life years; RNA: ribonucleic acid; SVR: sustained viral response; Tx: treatment.

[1] Cumulative number of cases over 2019-2030 and 2019-2035 periods, including those who were alive as of January 01, 2019 (i.e., at beginning of simulation). Percentages were calculated out of total CHC cases.
[2] Total CHC cases included diagnosed and undiagnosed F0-F4 cases, and those with ESLD.
[3] Diagnosed, i.e., tested RNA positive, including cases before developing ESLD.
[4] Individuals who initiated therapy and/or achieved SVR before developing ESLD.
[5] End-stage liver disease including decompensated cirrhosis, hepatocellular carcinoma, and liver transplantation.
[6] Costs represent total healthcare cost, excluding program implementation cost, rounded to nearest dollar and expressed in 2023 Canadian dollars.
[7] ΔQALY, ΔCost (incremental values) and ICUR were calculated relative to prior less costly strategy and excludes program implementation-related costs.
[8] The strategy was considered cost-effective if the incremental costs relative to prior less costly strategy were CAD <50,000 per QALY gained.

Supplemental Table S6. CHC care cascade and health and economic outcomes over 2019-2030 and 2019-2035 periods for >1965 birth cohort

	Predicted cases as of Dec 31, 2030[1] per 100,000 people			Predicted cases as of Dec 31, 2035[1] per 100,000 people		
	**Status quo**	**RNA rate = 98% and Tx rate=98%**	**RNA rate = 98% and Tx rate = 98% and AB testing rate x2**	**Status quo**	**RNA rate = 98% and Tx rate = 98%**	**RNA rate = 98% and Tx rate = 98% and AB testing rate x2**
**Care cascade outcomes**						
Total CHC cases [2]	1,218 (100%)	1,218 (100%)	1,218 (100%)	1,414 (100%)	1,414 (100%)	1,414 (100%)
Diagnosed at F0-F4 stages [3]	827 (68%)	896 (74%)	1,035 (85%)	988 (70%)	1,076 (76%)	1,228 (87%)
Initiated antiviral therapy [4]	459 (38%)	866 (71%)	1,001 (82%)	538 (38%)	1,041 (74%)	1,188 (84%)
Achieved SVR [4]	439 (36%)	823 (68%)	952 (78%)	515 (36%)	992 (70%)	1,134 (80%)
ESLD [5]	64 (5%)	39 (3%)	33 (3%)	97 (7%)	50 (4%)	41 (3%)
**Health and economic outcomes**						
Liver-related mortality, cumulative	37	23	20	63	33	28
Liver-related mortality in 2030, 2035	4.44	1.95	1.55	5.67	1.99	1.58
Life years per person	11.923	11.923	11.923	16.828	16.830	16.830
QALYs per person	9.510	9.511	9.512	12.883	12.886	12.887
Costs per person [6]	CAD 32,287	CAD 32,315	CAD 32,341	CAD 46,099	CAD 46,019	CAD 46,014
ΔQALY [7] per person	-	0.0015	0.0004	-	0.0030	0.0008
ΔCost [7] per person	-	CAD 28	CAD 26	-	CAD −80	CAD −5
ICUR [7] (sequential)	-	**CAD 18, 393**	**CAD 63,508**	**-**	**cost saving**	**cost saving**
**Program implementation's maximum cost for the strategy to remain cost-effective [8]**						
Cost per 100,000 people	-	**CAD 449,438**	**-**	**-**	**CAD 1,542,692**	**CAD 288,416**

AB: antibody; CAD: Canadian dollar; CHC: chronic hepatitis C; ESLD: end-stage liver disease; F0-F4: fibrosis stages, where F4 is cirrhosis; ICUR: incremental cost/utility ratio; QALY: quality adjusted life years; RNA: ribonucleic acid; SVR: sustained viral response; Tx: treatment.

[1] Cumulative number of cases over 2019-2030 and 2019-2035 periods, including those who were alive as of January 01, 2019 (i.e., at beginning of simulation). Percentages were calculated out of total CHC cases.
[2] Total CHC cases included diagnosed and undiagnosed F0-F4 cases, and those with ESLD.
[3] Diagnosed, i.e., tested RNA positive, including cases before developing ESLD.
[4] Individuals who initiated therapy and/or achieved SVR before developing ESLD.
[5] End-stage liver disease including decompensated cirrhosis, hepatocellular carcinoma, and liver transplantation.
[6] Costs represent total healthcare cost, excluding program implementation cost, rounded to nearest dollar and expressed in 2023 Canadian dollars.
[7] ΔQALY, ΔCost (incremental values) and ICUR were calculated relative to prior less costly strategy and excludes program implementation-related costs.
[8] The strategy was considered cost-effective if the incremental costs relative to prior less costly strategy were CAD <50,000 per QALY gained.

References

[1] Remis R. Modelling the incidence and prevalence of hepatitis C infection and its sequelae in Canada, 2007. 2011.

[2] Simmons B, Saleem J, Hill A, Riley RD, Cooke GS. Risk of Late Relapse or Reinfection With Hepatitis C Virus After Achieving a Sustained Virological Response: A Systematic Review and Meta-analysis. Clin Infect Dis. 2016;62(6):683-94.

[3] Micallef JM, Kaldor JM, Dore GJ. Spontaneous viral clearance following acute hepatitis C infection: a systematic review of longitudinal studies. J Viral Hepat. 2006;13(1):34-41.

[4] Erman A, Krahn MD, Hansen T, Wong J, Bielecki JM, Feld JJ, et al. Estimation of fibrosis progression rates for chronic hepatitis C: a systematic review and meta-analysis update. BMJ Open. 2019;9(11):e027491.

[5] van der Meer AJ, Veldt BJ, Feld JJ, Wedemeyer H, Dufour JF, Lammert F, et al. Association between sustained virological response and all-cause mortality among patients with chronic hepatitis C and advanced hepatic fibrosis. JAMA. 2012;308(24):2584-93.

[6] Planas R, Balleste B, Alvarez MA, Rivera M, Montoliu S, Galeras JA, et al. Natural history of decompensated hepatitis C virus-related cirrhosis. A study of 200 patients. J Hepatol. 2004;40(5):823-30.

[7] Sahakyan Y, Lee-Kim V, Bremner KE, Bielecki JM, Krahn MD. Impact of direct-acting antiviral regimens on mortality and morbidity outcomes in patients with chronic hepatitis c: Systematic review and meta-analysis. J Viral Hepat. 2021;28(5):739-54.

[8] D'Amico G, Garcia-Tsao G, Pagliaro L. Natural history and prognostic indicators of survival in cirrhosis: a systematic review of 118 studies. J Hepatol. 2006;44(1):217-31.

[9] Giannini EG, Farinati F, Ciccarese F, Pecorelli A, Rapaccini GL, Di Marco M, et al. Prognosis of untreated hepatocellular carcinoma. Hepatology. 2015;61(1):184-90.

[10] Charlton M, Seaberg E, Wiesner R, Everhart J, Zetterman R, Lake J, et al. Predictors of patient and graft survival following liver transplantation for hepatitis C. Hepatology. 1998;28(3):823-30.

[11] Wong WWL, Haines A, Wong J, Hamadeh A, Krahn MD. A province-by-province cost-effectiveness analysis and budget impact analysis of one-time birth cohort screening of hepatitis C virus (HCV) infection in Canada. Sci Rep. 2023;13(1):13484.

[12] Erman A, Everett K, Wong WWL, Forouzannia F, Greenaway C, Janjua N, et al. Engagement with the HCV care cascade among high-risk groups: A population-based study. Hepatol Commun. 2023;7(9).

[13] Jacobson IM, Lawitz E, Gane EJ, Willems BE, Ruane PJ, Nahass RG, et al. Efficacy of 8 Weeks of Sofosbuvir, Velpatasvir, and Voxilaprevir in Patients With Chronic HCV Infection: 2 Phase 3 Randomized Trials. Gastroenterology. 2017;153(1):113-22.

[14] Zoratti MJ, Siddiqua A, Morassut RE, Zeraatkar D, Chou R, van Holten J, et al. Pangenotypic direct acting antivirals for the treatment of chronic hepatitis C virus infection: A systematic literature review and meta-analysis. EClinicalMedicine. 2020;18:100237.

[15] Wilton J, Wong S, Yu A, Ramji A, Cook D, Butt ZA, et al. Real-world Effectiveness of Sofosbuvir/Velpatasvir for Treatment of Chronic Hepatitis C in British Columbia, Canada: A Population-Based Cohort Study. Open Forum Infect Dis. 2020;7(3):ofaa055.

[16] Xie J, Xu B, Wei L, Huang C, Liu W. Effectiveness and Safety of Sofosbuvir/Velpatasvir/Voxilaprevir as a Hepatitis C Virus Infection Salvage Therapy in the Real World: A Systematic Review and Meta-analysis. Infectious Diseases and Therapy. 2022;11(4):1661-82.

[17] An J, Park DA, Ko MJ, Ahn SB, Yoo JJ, Jun DW, et al. Direct-Acting Antivirals for HCV Treatment in Decompensated Liver Cirrhosis Patients: A Systematic Review and Meta-Analysis. J Pers Med. 2022;12(9).

[18] Tang W, Chen W, Amini A, Boeras D, Falconer J, Kelly H, et al. Diagnostic accuracy of tests to detect Hepatitis C antibody: a meta-analysis and review of the literature. BMC Infect Dis. 2017;17(Suppl 1):695.

[19] Yan J, Xie S, Johnson JA, Pullenayegum E, Ohinmaa A, Bryan S, et al. Canada population norms for the EQ-5D-5L. Eur J Health Econ. 2023.

[20] Saeed YA, Phoon A, Bielecki JM, Mitsakakis N, Bremner KE, Abrahamyan L, et al. A Systematic Review and Meta-Analysis of Health Utilities in Patients With Chronic Hepatitis C. Value Health. 2020;23(1):127-37.
[21] Government of Ontario. Ontario Drug Benefit Formulary/Comparative Drug Index, https://www.formulary.health.gov.on.ca/formulary/; 2023 [accessed 12 December 2023].
[22] Mendlowitz AB, Naimark D, Wong WWL, Capraru C, Feld JJ, Isaranuwatchai W, et al. The emergency department as a setting-specific opportunity for population-based hepatitis C screening: An economic evaluation. Liver Int. 2020;40(6):1282-91.
[23] Wong WWL, Haines A, Bremner KE, Yao Z, Calzavara A, Mitsakakis N, et al. Health care costs associated with chronic hepatitis C virus infection in Ontario, Canada: a retrospective cohort study. CMAJ Open. 2021;9(1):E167-E74.
[24] Krajden M, Kuo M, Zagorski B, Alvarez M, Yu A, Krahn M. Health care costs associated with hepatitis C: a longitudinal cohort study. Can J Gastroenterol. 2010;24(12):717-26.
